# Supplementary material for: The DJ-1-Binding Compound Exerts a Protective Effect in Both In Vitro and In Vivo Models of Sepsis-Induced Acute Kidney Injury
Source: Antioxidants (Basel). 2025 Jun 12;14(6):719. doi: 10.3390/antiox14060719 (PMC12189621; doi:10.3390/antiox14060719)
Supplement: Supplementary file 1 [file antioxidants-14-00719-s001.zip › antioxidants-3626155-supplementary.pdf]

DJ-1: therapeutic target in sepsis induced acute kidney injury

Zrufkó et al.

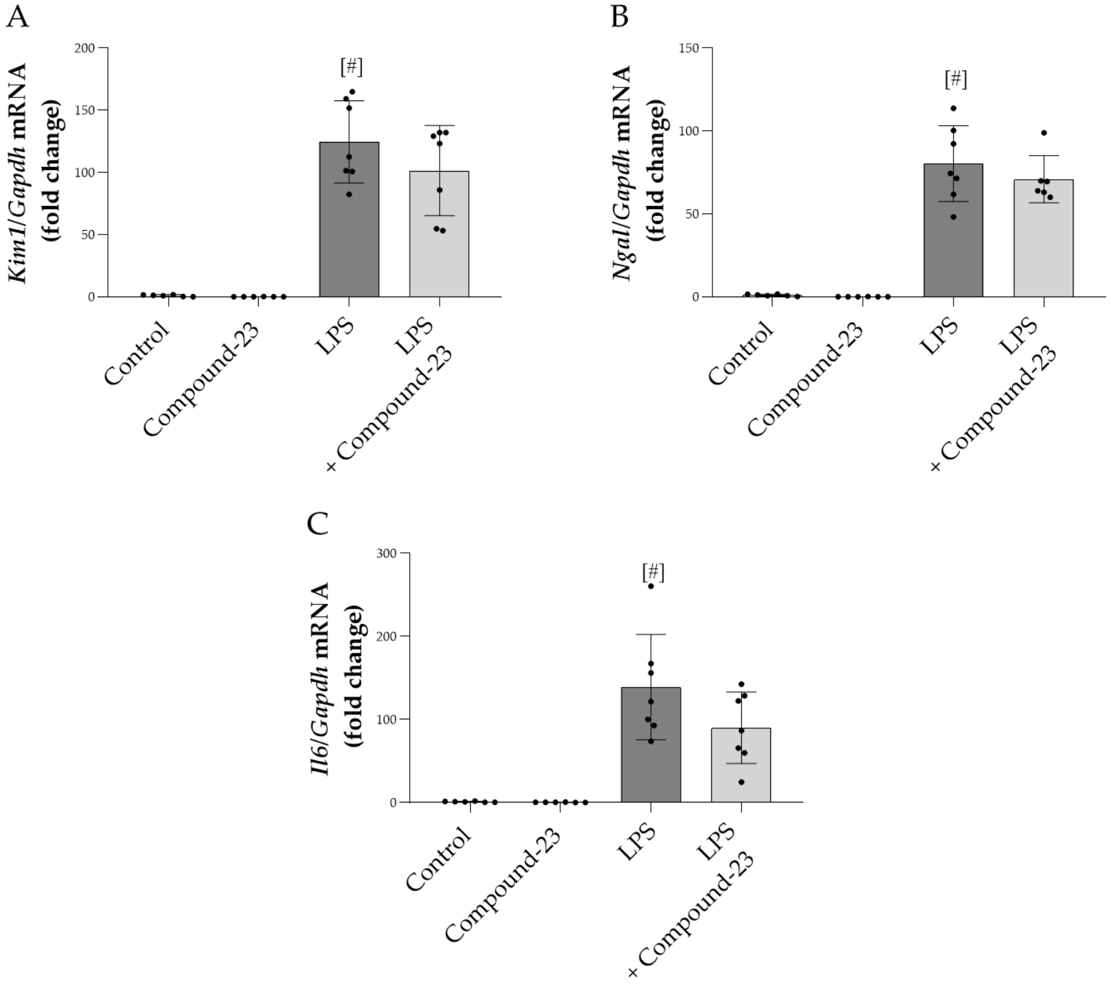

Supplementary Figure S1. Effect of DJ-1 binding Compound-23 (10mg/kg) on the LPS (10 mg/kg) induced acute kidney injury (AKI). The renal mRNA expression of *Kim1*, *Ngal* and *Il6* was determined by real-time PCR relative to *Gapdh* expression (A-C). Data were normalized and presented as the ratio of the mean values of the control group. Values were expressed as mean±SD. Dots represent individual values in each group (n=6–7); #p<0.05 vs. Control; ##p<0.05 vs. LPS.
